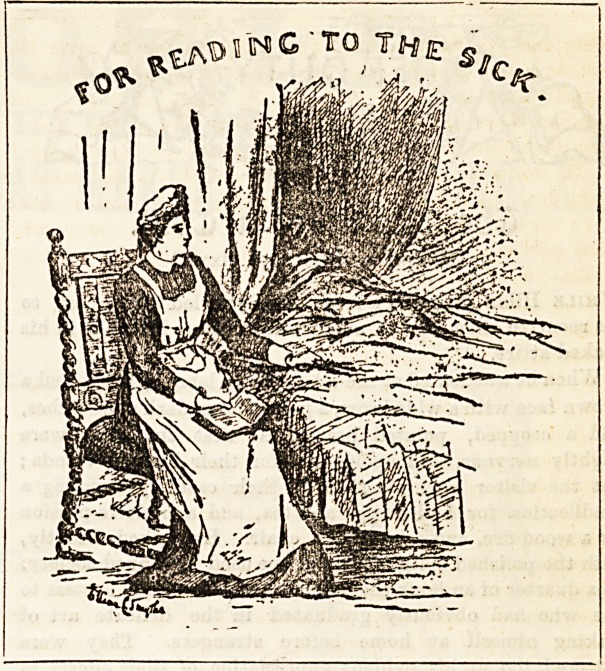# The Hospital Nursing Supplement

**Published:** 1892-09-24

**Authors:** 


### "The Hospital" Nursing Mirror.

Being the Extra Nursing Supplement of "The Hospital" Newspaper.

Contributions for this Supplement should be addressed to the Editor, The Hospital, 140, Strand, London, W.C., and should have the word "Nursing" plainly written in left-hand top corner of the envelope.

### En Passant.

DISTRICT NURSING AT CAMBERWELL.—We are pleased to hear of a staff of five nurses settled at Camberwell District Nurses' Home at the present date. In 1891, when the work was first started, there were only two nurses, who attended 213 cases. In 1892 there were 387 patients, and the visits paid during the year reached the large total of 13,215. Two probationers were trained, and having successfully passed their examinations, they received appointments as Queen's Nurses.

CLITHEROE DISTRICT NURSING ASSOCIATION was formed last November, and has a'ready a famous record of work well accomplished. Nurse Wade began her duties on February 1st, and by August 31st she had paid 1,739 visits to 160 patients. Some of the cases were very serious ones, and needed two or three daily calls. At first some of the people were opposed to the district nursing scheme, but it has now won for itself unanimous approval. The Ladies' Committee is to be congratulated on the success of the association.

BATHING AT SAN SEBASTIAN.—We read of the great heat in Spain, and the other drawbacks, but the luxuries which can be enjoyed there are less well known. At San Sebastian bathers can be conveyed to the sandy beach from the "Etablissement de Luxe" by a tramway, whilst those who prefer a "cabin" or machine can enjoy one of a far more commodious type than the English abominations. In this they must feel a certain amount of personal dignity, for it is drawn at a slow and stately pace down to the sea by a pair of beautiful oxen, those picturesque beasts which form a most ornamental feature in the local landscapes.

ROYAL FREE HOSPITAL, Gray's Inn Road.—With a few stray cases of cholera in England this year, and official warnings that a heavier invasion is to be anticipated in 1893, it is interesting to read the record of good work done in the past. In 1832 the R.F.H. threw open its doors to cholera patients, and 700 persons suffering from this disease were consequently admitted; and in '49 and '54 even larger numbers of sufferers entered and were treated. It is too busy and useful a hospital to need any "advertising" of its merits, beyond a bare record of facts which speak for themselves. But it is also a very poor little hospital, and, like the hundreds of workers who swarm within its reach, it goes on quietly paying its way, but "living from hand to mouth," and sorely needing external help to forward its urgently needed structural developments.

ANOTHER LADY LECTURER.—Jaeger's and other "woollen underwear" are of increasing popularity, but that does not make it very reasonable for a speaker at "cottage lectures" to tell her audience that health cannot be maintained without this luxury, &c., &c. Champagne and oysters, satin dresses and French boots are just about as easy of attainment for a labourer's wife who has struggled to bring up a hungry, healthy young brood on her husband's earnings of sixteen shillings a week. To be of any use, "homely talks" must be the outcome of sympathetic knowledge, and this is something far above book-learning or the acquirement of technicalities. Help, to make the best of what is within their reach, in sickness as well as health, is needed more than dissertations on Loofah and Jaeger, good though both may be for those who can obtain them.

ST. PATRICK'S HOME.—This is the first year in which the Home has worked in affiliation with Queen Victoria's Jubilee Institute, and the Committee are glad to say that not only has the nursing of the sick poor become much more extended in Dublin, but other towns have been supplied with nurses. Satisfactory work has been for many years success fully carried on by the St. Patrick's Home, and we are glad to find the affiliation has produced increased usefulness. Four probationers are to be annually trained for the purpose of becoming Queen's Nurses. The rules appear to be sensible ones, and the donations, Christmas gifts, &c., recorded in the report, are sufficient evidence of the interest taken by the public in the valuable department of district nursing.

NOURISHMENT FOR THE NEEDY.—The Middlesbrough Nursing Association seems to be doing valuable work in practical ways, for it not only provides skilled nurses for the sick, but nourishing dinners for the convalescent poor. Certain ladies undertake that one of them will on each day of the week supply a meal to patients whom the Lady Superintendent points out. Some one comes at the specified hour from the invalid's house, and shortly returns with a nicely-served and well-cooked dinner, which must be a valuable adjunct to the doctor's physic and the nurse's care. It sounds a pleasant and friendly way of helping needy neighbours, and must bring about a kindly sympathy between helpers and helped.

ABOUT HOSPITAL NURSES.—This is the heading of an article in the *Christian World* of September 1st, and the kindly common sense with which the writer treats the subject is quite refreshing. After the sentimental twaddle which people who know nothing about it constantly put into the columns of any paper which will accept their productions, it is really a bracing change to peruse such sentences as the following: "Selfishness makes its possessor an unfair judge, and selfish persons are really disqualified for hospital service. . . . Of course, nursing cannot be made play; it is too serious an occupation for that."

MIDWIFERY FOR NURSES AND WOMEN STUDENTS.—The Sisters of St. John have given up their maternity home at Battersea, and the work will now be carried on by the Clapham School of Midwifery. The in-patient branch (which numbered 150 cases last year) will be transferred to the Clapham Maternity Hospital, and the out-patient work, which averages 650 patients per annum, will be under the general superintendence of Dr. Annie McCall and the Clapham School of Midwifery. This new Battersea branch will be under the special charge of Miss Madgshon, M.B.Lond., who was, for over a year, house surgeon at the Clapham Maternity Hospital subsequently to passing a post-graduate midwifery course at Vienna. Miss Madgshon, the senior medical officer, will live at 18, Albert Road, Clapham, with the junior medical officer, Miss Annie Anderson, M.B.Lond. These ladies will be assisted by a staff of students, who may arrange to attend out-patients only or else to do in-patient work as well at the Clapham Maternity Hospital. Nurses wishing to become certified midwives can learn all particulars on application to Dr. Annie McCall, 131, Clapham Road. The Clapham Maternity Hospital has already received 415 cases, and the large number of 2,500 patients, with only one death, have been treated by the midwifery school. Such a record, indeed, speaks well for the work done, and it also gives good promise for the "new departure" at Battersea.

### Lectures for Asylum Attendants.

By William Harding, M.B.

### II.—VENTILATION.

To attend to the ventilation of her ward is one of the most important duties of a nurse and one which requires constant attention. Perfect health cannot be maintained without a plentiful supply of fresh air, and it is vain to attempt to restore the feeble and debilitated without it. The object of ventilation is to provide this fresh air and to get rid of that which has been breathed and rendered impure. To the nurse personally this is all important. The constant breathing in of foul air will soon pull down her strength and sap her energies. Headaches, loss of appetite, anæmia, and even phthisis may result. Impaired health and irritability of temper will certainly follow, and under such circumstances a nurse can neither do justice to herself nor to those under her charge. If she do not accustom herself to look to the ventilation as a matter of routine it is very likely to be overlooked, and she will have the mortification of hearing those entering her ward remark upon the closeness of it. The feeling of stuffiness, which may be used as a practical test that the ventilation is insufficient, is not apparent to anyone who has been in the ward for some time, but at once strikes an individual who has just come from the fresh air outside. To breathe in previously respired air is slowly to commit suicide, and this form of self-poisoning is one of the most unpleasant ways of affecting that end.

Air consists of a mixture of gases—oxygen, nitrogen, and carbonic acid—the last in very small amount. The oxygen and the carbonic acid are the important constituents. The nitrogen plays no part in respiration, but merely dilutes the other gases. Oxygen is taken from the air in the lungs, and carbonic acid with some water and organic matter is returned to it. It is this organic matter which causes the unpleasant stuffiness in a close ill-ventilated apartment. The air also takes up water from the sweat of our bodies, and is tainted by smells and impurities from various sources as from gas escapes, &c. It also becomes loaded with particles of dust, and in sick wards this is a very important element. Some of the insane emit strong and very unpleasant odours from their skins; others, again, owing to their dirty habits, soon pollute the atmosphere of a whole ward if not immediately looked to. Escapes of gas are very dangerous. Fortunately its well marked smell at once attracts notice to it.

The air which leaves the ward is poorer in oxygen than it was when it entered, but contains more carbonic acid, water, organic matter, and other impurities which it has gathered. The chemical standard of impurity is the amount of carbonic acid, but a practical guide is the feeling of stuffiness perceived on coming in from the fresh air. The ideal to be aimed at is to keep the air inside as pure as that out of doors, and to effect this without causing perceptible draughts. This is not easy to manage even in a room filled with the sane, who ought to be able to appreciate the necessity for, and to aid in maintaining proper ventilation; but among the insane it is often very difficult. The perverse obstinacy of some patients is very trying. They seem to think that an open window is a positive danger, and must be closed at once.

There is always some change taking place in the air of a room. The air outside mixes with that inside through the pores of the walls, the chinks in the windows, &c. The warmer and lighter air inside also tends to rise and escape, while its place is taken by the colder and heavier air which enters from without. The fire causes a rush of warm air up the chimney, and creates a draught up it, which assists materially in ventilating the room. In an ordinary apartment with only three or four persons in it these means are effective enough, but are not sufficient for a large room with a number of inmates. Under the latter circumstances intelligent use must be made of windows and special openings in the wall called ventilators. With other and more elaborate methods the nurse would not be concerned. The points for her to keep in mind are :—

1. To see that the means for ventilation are in working order.

(*a*) Ventilators. These are very liable to get blocked. Even when the current of air can be proved to be passing from within outwards many patients will declare that they are being killed by the cold draught, and if they possibly can do so will plug up the gratings. Dust and fluff are apt to accumulate and interfere with the free passage of air. Those openings on a level with the floor may be used as receptacles into which dust may conveniently be swept, and some patients appear to believe that their only use is to afford convenient hiding places for odd slippers, stockings, &c. Obstructions may also be caused by materials which get in from outside.

(*b*) The chimneys should not be blocked, and should be kept free from accumulations of soot.

(*c*) The windows will require intelligent attention, as by proper management even on very windy days they can be made available, and in most wards by their means cross-ventilation or a stream of air passing across the ward can be obtained. A close watch will be necessary, as many patients are ready to shut the window whenever opened. Of course, the windows of single rooms and dormitories should be thrown open as soon as the patients have left them.

(*d*) Doors should not be used as a means of ventilation. As a rule, to do so only means admitting air which has been already polluted more or less.

(*e*) Any dampness of the walls should be at once reported. It interferes with the passage of air through the pores in the wall; affords a resting-place for dust; keeps down the temperature of the ward, and also provides places in which infectious germs may develop.

2. To prevent inside impurities.

(*a*) Dust must be removed. Particles from skin, hair, clothing, &c., become deposited on the furniture and walls, and with them some of the offensive organic matter in the air. In the wards for the sick special attention should be paid to this.

(*b*) Some of the patients will be found to give out very offensive odours from their skin, and will require particular care as regards washing and general cleanliness.

(*c*) Wet and dirty cases if neglected will soon pollute a whole ward. Patient endeavours to inculcate better habits will do much, and the bathing and changing when required must be done at once.

(*d*) The foul linen should be at once conveyed to some place where it cannot pollute the air within the building. In sick wards, chambers, &c., should be emptied and cleansed at once.

(*e*) Lavatories and water-closets should be kept scrupulously clean, and inspected by the charge nurse regularly and frequently. They should be well ventilated, and the windows in the passage that leads from the ward to the closets should be kept open when possible, and the door that cuts off the closets from the ward kept closed. For purposes of observation this door is generally furnished with large glass panels. It is important to notice the under-surface of the seats in the water-closets. In the male wards the urinals are often a great source of impurity. Sinks should be kept very clean, and their traps in order, and any escape of gas should be at once reported.

(*f*) Where the heating is effected by steam disagreeable smells are sometimes caused by the dirty practices of mischievous patients who foul the steam chests. Some patients also have a habit of drying wet and filthy rags on the heating apparatus.

(g) The single rooms and dormitories occupied by dirty patients will give much trouble, and will require careful cleansing with hot water and Jeye's fluid or some similar preparation. The nurse should be careful to see that the floor is well dried after being scrubbed. She should also notice that the ventilators are not daubed with filth, and that the cracks between the boards of the floor, doors, and wall (if boarded) are clear.

She must carefully supervise the patients who are assisting her. They will sometimes begin to scrub out a clean room with dirty water and cloths. The result is not beneficial to the sweetness of the dormitory or corridor.

The value of sunlight should not be forgotten, and in order to gain its full benefit the windows should receive regular attention. Each time the charge nurse enters the ward she should be on the outlook for stuffiness, and should ask those under her who have been absent from the ward for a while to report on their return whether they notice any closeness or smells.

### London's End.

It seems the end of everywhere—there is such an unfinished and disjointed air about the whole place. The roads are unmade, and bordered on each side with a ditch of water, and they mostly end nowhere. The houses are in patches in the middle of potato fields and common land; they are real mushroom houses, many of them, without any foundations. You see some cartloads of bricks and some ready-made doors and windows shot down on a vacant space, and next time you pass you see half a dozen houses—all occupied. London repudiates this tag-rag town of one hundred thousand inhabitants, and yet it joins London, it is the latest extension eastward, down past the docks into the marshes of Essex.

It is Plaistow which is at present London's end—Plaistow, with its row after row of two-storey houses; Plaistow, with its rambling outlying parishes; Plaistow, where fever flourishes and cholera is to be dreaded. You pass through criminal Whitechapel, through crowded Shadwell, to emerge on the flat, bleak land where Plaistow grows. The only breaks to the flatness are the masts of the ships in the river to the south, and a cluster of huge gasometers to the west. Plaistow is not crowded, it has a quiet, almost forsaken look, desolate in the extreme. Until you have wandered for miles and miles along its incomplete and melancholy streets it is impossible to grasp the fact that it contains a hundred thousand inhabitants. This huge excrescence, as it were, of East London, built on marshy ground where fever flourishes, has no fever hospital. In one house we entered there was a mother and her few hours' old baby in the same room with three children peeling after scarlet fever. The Plaistow fever cases do not come within the Metropolitan Board's returns, so few people grasp the fact of how fever flourishes, and is callously treated in ordinary houses. There is a hospital at Plaistow, a great big building eminently fitted for isolation, but at present it is rented by rich Kensington, and formerly it stood empty and unused. One wonders, since the authority of the London County Council does not extend so far east, if Essex cannot boast a county council capable of grasping the difficulties of London's end and dealing with them efficiently. Cholera would find a fine field at Plaistow, and there are absolutely no means by which it could be grappled with there. The home of the gipsies is at Plaistow—the real gipsies, the Lees and the Smiths, have their head encampments on the vacant grounds. There are made caravans, and in case anyone wishes to take a fashionable holiday in one, as the Duke of Newcastle is now doing, we may mention that one of the Smiths told us that the cost of a new caravan for one horse was from £40 upwards.

We have remarked before in these pages that the trained nurse is now ubiquitous—we came across her amongst the gipsies. There drove up on the grass a dilapidated pony trap, drawn by a jogging little grey pony of the roughest and slowest description, and a nurse in cloak and veil threw the reins on the pony's back, got out, and stooping low, entered one of the tents. We followed her. She was squatting (for it was impossible to stand upright, and there were no seats to sit on) beside a bed where lay a mother and twins. The little low tent was frightfully hot, the flies in hundreds buzzed round the mother and children, and we retreated quickly to the open air. "One of the nurses from St. Mary's Home," explained the father of the twins, a fine looking man, speaking excellent English, and with none of the twanging accent or slouching appearance of a Whitechapel rough; in fact, a real gipsy, and not a tramp. The nurses of St. Mary's seem to be the one attempt at healthiness in Plaistow. In winter, though wet to the knees with snow, which is never cleared away, and pierced through and through with the cold wind from the marshes, they tramp about this great, lonely district, tending the sick in their own homes; or, if the cases are very bad, moving them to their little cottage hospital and treating them there. Sister Katherine, who is at the head of St. Mary's Home, went to Plaistow with one fellow-worker only four years ago, but the work was enormous, and Sister Katherine rose to the occasion. Now there are something like twenty nurses, a nice little hospital with an out-patient department, a crêche, and all sorts of other helps to health. But a fever hospital it is beyond the powers of the nurses to supply, and so London's end, lonely and desolate, pleads for greater interest in its needs, that the voice of the public may demand for it what it apparently cannot get for itself.

### How to become a Dispenser.

This is a question so often asked and so seldom adequately answered, that a few suggestions as to the studies needed and the kind of examinations which have to be passed, may prove helpful to the readers of The Hospital. The post of assistant dispenser in a country hospital is one peculiarly suitable to a nurse who has had the necessary training to qualify her for holding it. For this two courses are open—the first and simplest is to pass the examination of the Society of Apothecaries for "A certificate of qualification to act as an assistant in compounding and dispensing medicines." This enables a person to go as assistant to a chemist, or in a hospital; that is to say, to dispense under supervision, according to the Pharmaceutical Chemists Act; but not to sell poisons, nor to make up prescriptions which contain them, nor, of course, to set up a shop where he or she would be solely responsible. The second plan is to pass the minor examination of the Pharmaceutical Society, and then become a fully-qualified chemist. The examination held by the Apothecaries' Society is not a difficult one, but a thorough knowledge of materia medica, as contained in the "British Pharmacopœia," both organic and inorganic, is necessary.

In addition to this, botany must be mastered, especially in regard to medicinal plants; a knowledge of chemistry is required to enable the candidate to understand the Pharmacopœia. Theoretical and practical "pharmacy" will need careful study which can be partly supplied by books, but also needs actual and repeated practice. For those who live in London this is easy, as there are various schools of pharmacy where full instruction is given, such as the South London School of Pharmacy, 325, Kennington Road, and Middlesex College of Pharmacy, 40, Charlotte Street, W.; the latter is convenient for nurses, as they can pay down a fixed and inclusive fee to cover the whole course of study, and this allows of repetition until the examination is passed without any limit of time. This enables those who cannot attend every day of the week to continue their preparation during a greatly prolonged period.

In towns where these schools do not exist, the lessons in practical pharmacy must be obtained from a local chemist. We append a list of books which will be found useful, with the published prices, from which the reader can deduct the usual discount of 3d. in the shilling: "Morris' Class Book of Inorganic Chemistry" (published by Philip and Son, 32, Fleet Street, price 2s. 6d.); "Lessons in Elementary Botany," by Professor Oliver (Macmillan and Co., price 4s. 6d.); "Bentley's Materia Medica" (Longmans, price 7s. 6d.); "Bentley's Structural Botany" (Churchill, 7s. 6d.); "British Pharmacopœia" (Spottiswoode, 6s.); "Ince's Latin Grammar of Pharmacy" (Baillière, Tindall, and Cox, 5s.); and "Muter's Materia Medica" (Simpkin, 12s. 6d.)

The examination is held on the fourth Wednesday of every month, and a candidate who fails must wait three months before trying again. The fee is two guineas for the first, and one guinea for each re-examination, should such be unfortunately necessary, and intending candidates must give notice and pay the fee seven days in advance. The syllabus and all further details regarding this examination can be obtained by writing to Secretary, Apothecaries' Hall, Blackfriars, London. The following is an extract from the syllabus: "The examination consists of two parts; (a) practical, the compounding and dispensing of medicines; (b) oral; translation of prescriptions, the materia medica, pharmacy, chemistry, and botany of the British Pharmacopæia."

For the minor examination of the Pharmaceutical Society, much more is required. A candidate must be twenty-one years of age, and must pass a preliminary examination in the following subjects:—

I.—Latin. Grammar, translation of easy sentences from English into Latin; translation either of Cæsar's "De Bello Gallico," Book I.; or Virgil's "Æneid," Book I.

II.—English. Grammar; composition.

III.—Arithmetic. First four rules; simple and compound; vulgar and decimal fractions; simple and compound proportion; and a thorough knowledge of the British and Metrical System of weights and measures.

This examination is held quarterly by the Pharmaceutical Society, in January, April, July, and October, in London and other centres; but a certificate of having passed any other examination in arts, viz., College of Preceptors, Apothecaries' Society, &c., will be accepted instead. A list of the centres and of these examinations is given in the syllabus, which can be obtained from the Secretary, Pharmaceutical Society of Great Britain, Bloomsbury Square, London.

Before presenting herself for the minor examination, the candidate must have dispensed for three years and acquired a thorough knowledge of pharmacy and a practical acquaintance with the methods of preparing the various drugs used. She must understand practical dispensing and be able to read prescriptions, she must know materia medica, botany, theoretical and practical chemistry, and physics. A very detailed account of the requirements under each head is set forth in the syllabus. The fees are: Preliminary examination, two guineas; minor, five guineas, and for re-examinations in case of failure, one guinea and three guineas respectively. For the minor the following additional books will be found useful: "Attfield's Chemistry" (Van Voorst, Paternoster Row, 15s.); Dr. Muter's "Short Manual of Analytical Chemistry" (Simpkin and Marshall, 6s. 6d.)

These are the chief points respecting the books, studies, &c., which are necessary for the aspiring dispenser. In cottage hospitals "a nurse who can dispense" is sometimes required, and a thorough knowledge of drugs must under all circumstances be of considerable value to the student.

### Everybody's Opinion.

*[Correspondence on all subjects is invited, but we cannot in any way be responsible for the opinions expressed by our correspondents. No communications can be entertained if the name and address of the correspondent is not given, or unless one side of the paper only be written on.]*

### HOLIDAY HOMES FOR NURSES.

A correspondent writes to remind us of a pleasant home on the Sussex coast. When London was enveloped in fog last winter the little town of Bognor was favoured by continuous fine days and brilliant sunshine. Terms and particulars will be given by Miss Harrison, Lady Superintendent, 2 and 3, Denmark Terrace, Bognor.

### "MASTER MARINER."

Nurse R. desires to thank the writer of the article in The Hospital of August 27th, headed "A Warning." She was left by a patient "in a strange land," she says, "without friends," and a servant's ticket for her return journey. The "Master Mariner" inquired into the circumstances, and eventually secured for our grateful correspondent "a happy and comfortable voyage home." All nurses are indebted to this gentleman for his kind championship of their interests.

### BELSEY v. McLELLAND.

"Personal Knowledge" writes: If you will find space for this letter I shall be very grateful. In the Nursing Supplement to The Hospital of July 30th there is a brief account of the case "Belsey v. McLelland," recently tried in the Birmingham County Court. Towards the close of the account it is stated that "the jury naturally enough found that there was no assault and no libel, and judgment was given for the defendant." To those acquainted with all the facts of the case this finding of the jury was not so much a matter of course as the words quoted seem to imply. Even Miss McLelland, the Matron herself, practically admitted the assault by paying 10s. into court in satisfaction of the damages. And, with regard to the libel, she stated in court, of the letter in which it was alleged that the libel was contained, "I am very sorry that I wrote it." Miss Belsey had undoubtedly a very strong case, but she laboured under two disadvantages. One was that she had to fight against a local institution, that is, an institution of the district in which the trial took place, and one of a kind for which people generally have a good deal of sympathy. She was also placed at a disadvantage through those who had the legal management of the case, relying too much upon the facts which told in her favour, without taking the trouble to get them substantiated in court by witnesses. If witnesses had been subpœnaed from amongst the nurses of the infirmary to describe the condition of Miss Besley immediately after the scene during which the assault was said to have been committed it would have been clear that the matter was much more serious than it was made to appear during the trial. And this would have been made still more clear if the doctor, who was called in on her reaching home, had not been prevented from giving evidence at the trial by having on that day to sit for an examination. Besides having a passion for the work, Miss Belsey has exceptional capabilities for the calling of nursing, to which, of her own free choice, she had devoted herself, and she would not have been turned out of the path in which she was hoping to receive the equipment for it by anything of a trifling nature. In commencing the action against Miss McLelland, Miss Belsey was not actuated by either vindictive or mercenary motives. All she wished to obtain was some reasonable compensation for her seven months' service and the expense to which she had been put. She was also sanguine enough to hope that her action—whether she won her case or lost it—might possibly lead to results that would be beneficial to probationers generally, whose lot is admitted to be a hard one, and who while suffering are, as a rule, obliged to suffer in silence. It seems to be the law of life that the path by which blessing comes to the many must be for somebody the path of suffering. The sufferer in this case is a refined, gentle-spirited, patient, self-denying woman, whose highest pleasure in life consists in serving others who, by a cruel fate, is driven from an avocation for which she was and is pre-eminently fitted, and to which she was enthusiastically devoted. P.S.—I have omitted to state that Miss Belsey was never off duty for a single day through sickness during the whole seven months of her residence at the Worcester Infirmary.

ROYAL NATIONAL PENSION FUND FOR NURSES.

"THE SUPERINTENDENT OF THE LEEDS TRAINED NURSES' INSTITUTION" writes: I was present at the meeting at the Leeds Infirmary, on September 14th, when Mr. Burdett, the founder and deputy-chairman of the Royal National Pension Fund for Nurses, addressed nurses and others interested in nurses, on the advantages to be derived from joining this Fund. From the first institution of the Royal National Pension Fund for Nurses, I have been a great advocate of the claim that this Fund has on the thoughtful consideration of all women engaged in nursing. Ten of our nurses belonged to the first 1,000 nurses who joined the Fund. It must of necessity present unprecedented advantages. It was founded through the munificence of several of our merchants, and since its foundation the funds have been still further augmented by a generous public. It has been suggested that the premiums required are high for the pensions absolutely promised, but it is surely more desirable to establish this Fund on a business-like and secure basis, and augment the minimum pension promised from the bonus fund. Mr. Burdett stated that any nurse paying for a pension of £15 a year, may expect to receive £26 a year when she draws her pension. I have such a high opinion of the Royal National Pension Fund for Nurses that I should like to know every nurse in the United Kingdom belonged to it, but I differ from Mr. Burdett in the way he wishes this to be accomplished. He suggests that the committees of hospitals and institutions should come forward and pay half a nurse's premium. We consider it preferable to encourage our nurses to join the Fund without this present help which, with the adequate salaries given, we consider them able to do. The method of helping them we prefer to adopt, is to pay a certain sum yearly into the Fund to be used for the augmentation of our nurses' pensions when they become due. We have done this because we think it important not to destroy that spirit of self-dependence and prudent foresight, which every woman should possess. Personally, I should be glad if hospital authorities would adopt a similar method for helping those nurses who have rendered them long and efficient service, because I think such service deserves even more recognition than the payment of a very adequate yearly salary. I earnestly hope that many more nurses will now join this Fund, after hearing from Mr. Burdett the many advantages that must accrue to them by doing so.

### Notice to Nurses.

In answer to many inquiries, we are glad to say that nurses can now obtain "How to Become a Nurse," by Honnor Morten, price 2s. 6d., at *Scientific Press* Office, 140, Strand.

### Appointment.

MISS MARGARET ALICE LAYCOCK, who has been appointed Matron of the Bromsgrove Cottage Hospital, was trained at the Royal Southern Hospital, Liverpool, where she afterwards held the post of staff nurse for three years. Miss Laycock then became Matron of the Manchester Ship Canal Hospital, Ellesmere Port, where she did excellent work for three and a half years. She has very good testimonials, and we wish her every success in her present post.

### Cholera Lectures.

THE lecture-room at the Midwives and Trained Nurses' Club was crowded on Friday evening last, when Dr. Duncan gave a lecture on "Cholera," a subject which his Indian experience specially fitted him to speak on.

HOME.

"I WANT to go home," says the little child, sad and lonely, though surrounded by playmates and kind friends. It misses its parents, and the feeling of security which goes with them, so it renews it's cry for "home," and refuses to be comforted.

"Home, sweet home," murmurs the young sailor when far from his native shores. He had despised what he called the humdrum pleasures of his father's house, and had run away from home to be free and see life. Now that he has gained his desire, an inexpressible longing for what he has lost comes over him, and he counts the days till he shall again be clasped to his mother's breast.

"I want to go home," says the old man who has come to fourscore years. He has been fairly prosperous, still the changes and chances, cares and trials of this mortal life have proved to him that here we have no abiding city. But he has sought one to come, and his happiness now consists in dwelling on the thought of that land far away, where saints in glory stand, and to which his weary feet are hastening.

Yes, "There is no place like home." There we store our treasures, there our loved ones dwell; the very word is sweet to our ear, it breathes of security, peace, comfort, and repose. The sufferings of the sick are sometimes aggravated by their desire for home, when circumstances have caused them to be carried to a hospital bed, and though they would lack the care and skill there lavished on them if they went back to their own homes, yet they continually say, "Home is home, be it never so homely." And in a great measure it is true, for the love of home is deeply seated in every heart, and this unreasoning yearning to return from whence we came, points plainly to the fact that we are but strangers and sojourners here on earth, and our natural desire for protection, ease, comfort, freedom, which is comprised in the one word home, will only gain its full fruition in the mansions prepared for us by our heavenly Father. Our earthly homes, even when happy and full of love, are but types of that better one far out of sight, of which we dream, of which we sing.

Let us then, dear sick friends, grasp the promised comfort given us in our Lord's word, "I go to prepare a place for you." To know that when we pass from this world of sin and suffering we have a better one to go to, that

There is a blessed home
Beyond this land of woe,
Where trials never come,
Nor tears of sorrow flow, is a hope which we cannot afford to throw away. Let us follow in our Saviour's footsteps. This was a path of daily toil and woe, but it led to glory. Now He sits on God's right hand, ready to welcome us into His Kingdom. Now He shows His hands and His side, and makes intercession for our sins. Lord Jesus bring us from our trials and sufferings to that Home where endless days shall be spent in everlasting light and glory.

### The Result of a Chill.

(*Continued from page* clxxx.)

While Hilda prepared a fresh tea, Sybil led their guest to his room to allow him to make what change he could in his soaked attire.

When he was seated at the tea table the lamp light showed a brown face with a wide brow, a pair of long, dark moustaches, and a cropped, pointed beard. At first the girls were slightly nervous, and apologetic for their homely viands; but the visitor soon put them at their ease by averring a predilection for boiled eggs and tea, and a positive passion for a wood fire, and a hammock chair. He talked fluently, with the polished sprightliness of one used to refined society. In a quarter of an hour they were charmed to play hostess to one who had obviously graduated in the delicate art of making himself at home before strangers. They were flattered, too, at his evident appreciation of their efforts to make the old room artistic and comfortable by a hundred little devices. He admired the rugs, the vases, and the quaint Chinese sketches on rice-paper, and tenderly handled an oriental plate, while he descanted upon ceramic art with the rapture of a *dilettante*.

"Do you paint?" he asked, as his gaze fell upon a canvas.

Sybil confessed to following art as an avocation. Upon hearing this his face expressed some slight pleasure. Asking permission to inspect the picture, he held it to the light for several moments.

"Near here?" he asked.

"A study in this valley," returned Sybil, flinching at his fixed scrutiny of her work.

"A charming subject," he observed. "Done in a good light, too."

"At sunrise," she said.

"Yes. May I criticise it?"

"Oh, certainly," Sybil replied, demurely.

"You go direct to nature, I see," he began.

"Of course. Should not every artist?"

"Yes, by all means," he answered, with a little smile. "Go to nature as much as possible, study her under every aspect and condition; live with her, like Turner, but don't copy her exactly."

Sybil darted a look at him which expressed revolt at this injunction. He noted it, smiled again, and set the picture down.

"Doesn't one of the principal critics of the new school, Mr. Lancaster, commend a close adherence to nature?" she asked.

"He does. But he certainly deprecates a photographic baldness of style."

"Then you think I am too rigid in my fidelity to nature?" She hung on his answer, with no satisfactory reason for doing so, unless his dominant manner vaguely savoured of acumen.

"If I may say so—yes, I do," was his answer. "You draw admirably; you have selective judgment, but you lack colour. Still, I am very pleased with that sketch, because it shows undoubted promise. You will pardon me for my plain speaking."

"Oh, yes; I am much obliged. It is gratifying to hear that one's work is not utterly thrown away."

Perhaps the light tinge of irony that she infused into her tone disconcerted the visitor, for he made no more comments upon art for the time being. He sat down, and talked of the supposititious Druidical remains of the district, the varied beauty of Devonshire scenery, the last play at the Lyceum, and finally drew the girls into a disclosure of their main reasons for leading a life of seclusion.

"How delightfully practical, and yet how idyllic," he said. "Were I not a slave to necessity, I should love to sequestrate myself in a wild part like this, and simply walk about or read the books one finds no time to read in town. I think I would sacrifice half my income to do that. It's a lazy programme, though. You work hard."

"We have our livings to earn," said Hilda.

A lamp with a yellow shade threw a soft, amber light on Sybil's oval face, and while she talked the stranger's keen glance was fixed upon her. Plainly enough his gaze evinced to Hilda a mixture of curiosity and admiration. She discerned, too, that Sybil was much interested in their guest, for when his look was directed at the blazing faggots she watched his profile from beneath drooping eyelids, and appeared to be studying his features as she would have studied a fine portrait. Considering the shortness of their acquaintance, it was strange to Hilda that they should have made such quick progress in friendliness. The tourist was almost fraternal by midnight, and, as she said afterwards, when he shook hands before retiring, it seemed as though they had known each other for months instead of hours.

(*To be continued.*)

### Where to Go.

The next lecture at the Midwives' Institute and Trained Nurses' Club will be given on Friday evening, September 23rd, at a quarter to eight, by Rowland Humphreys, L.R.C.P., Lond. Subject: "The Feeding of Children and Invalids." A few tickets to non-members 6d. each.

Science Lectures at same hall on Tuesday evenings. Hon. Manager, Miss Cons, Morley Memorial College.

Organ Recitals at Royal Albert Hall every Sunday afternoon, at three o'clock. Admission free.

Saturday Evening Lectures, at Working Men's College, Great Ormond Street, will commence October 8th, at half-past eight.

Gresham Lectures, at Gresham College, Basinghall-street. "Physic," by Dr. Symes Thompson, at six o'clock, on October 4th, 5th, 6th, and 7th.

University Hall, Gordon Square.—Ten lectures on "The English Citizen, Past and Present," by Graham Wallas, M.A., at eight o'clock, on Thursday evenings, commence October 13th.

Lectures for Nurses at Glasgow.—We are glad to read that Dr. Lindsay Stevens is giving a course of lectures on "Cholera" to the Glasgow Sick Poor and Private Nursing Association.

### Notes and Queries.

### Queries.

*Eastbourne.*—Can anyone recommend a pleasant holiday home near Eastbourne for a nurse who needs rest.—*Nurse Bee.*

*Male Nurses.*—Can any of your readers give a list of hospitals or institutions where male nurses are trained.—*W. A. H.*

### Answers.

*Epileptic* (*M. L. P.*)—Thank you for letter.

*Specialist* (*Nurse*).—We do not prescribe.

*Dispenser* (*Scotia*).—Read our rules, to which we *strictly* adhere. Name and address are always indispensable. We give the information you ask for in another column.

*Probationer* (*Charlotte*).—You have overlooked rule 5, and omit your name and address.

*Dispensing* (*Etta*).—See article in this week's "Mirror."

*Cholera* (*Nurse Joan*).—You will find Dr. Duncan's lectures fully reported in "Nursing Notes" for October.

### Wants and Workers.

A district nurse appeals for old night-gowns, and also for worn-out linen, for the use of a sufferer from cancer.—Address *Nurse M.*, 82, *Victoria Street, Ashton-under-Lyne.*

## Figures and Tables

**Figure f1:**